# Research and Remedies

**Published:** 1912-05-04

**Authors:** 


### RESEARCH AND REMEDIES.

### More Work on Sleeping-Sickness.

The first report—a preliminary one—of the Commission sent out to Northern Rhodesia by the Liverpool School of Tropical Medicine contains a series of facts which have been ascertained to hold good for the particular region where the Commissioners are at work. In the Luangwa Valley, states their interim report, the human trypanosome is transmitted by the tsetse fly, *Glossina morsitans*. Almost 5 per cent. of the fly population of this species are found to be infected and capable of transmitting the parasite. It is found that after an uninfected fly has a meal off an infected animal, about fourteen days elapse before that fly becomes itself infective; but once it has reached this stage the infectivity persists for the rest of its natural life, and is always active. Mechanical transmission does not occur if a period of twenty-four hours has elapsed since the infecting meal. Some evidence exists which is believed to show that in the interval between the infecting meal and the date at which transmission becomes possible, the parasites in the flies are non-infecting. This is a curious observation, the exact significance of which is at present obscure; but even more astonishing is the finding of the human trypanosome in four distinct species of antelopes and in a native dog. The complicity of the crocodile in the spread of sleeping-sickness was suggested by Koch, and the buffalo has been likewise incriminated by Selous and others; but the whole ungulate tribe and every domestic animal now seem to fall under suspicion as a result of these investigations.

### A Substitute for Oil of Turpentine.

The disadvantages and the dangers associated with the use of oil of turpentine have led to the search for a substitute which shall be an efficient disinfectant for the air-passages and at the same time be without any deleterious action on the kidneys. A preparation known as eulimen, derived from oil of lemons, has obtained considerable favour in Germany. It is a colourless, limpid, vegetable oil, chemically identical with limonene, which is one of the terpene group of hydrocarbons, having the formula $C_{10}H_{16}$ and a boiling point of 175°C. Several articles have already appeared in the German medical journals pointing out the usefulness of this oil in the treatment of bronchiectasis and purulent bronchitis. Professor Kobert, of Rostock, was the first to draw attention to the excellent results obtained with eulimen as a disinfectant for purulent and foul-smelling expectoration. A paper by Dr. Goliner appearing in the Vienna *Aerztliche Central Zeitung* gives further favourable reports on this preparation of limonene in cases of chronic pulmonary catarrh, bronchiectasis and fetid bronchitis. The volume of the sputum is rapidly diminished and its foul odour quickly destroyed. The method of administration is the same as that usually employed with oil of turpentine—namely, by inhalation of the vapour obtained by pouring a small quantity on to boiling water or by administering it internally in doses of from ten to twenty drops on a lump of sugar.

### A New Nervous Disease.

Dr. S. A. K. Wilson, Registrar to the National Hospital for the Paralysed and Epileptic, describes a new form of nervous disease which he names progressive lenticular degeneration. It occurs in families; is always associated with cirrhosis of the liver, though the latter may be quite unsuspected during life; and is always fatal, so far as experience of it has yet revealed. The pathology of the disease is a bilateral symmetrical softening of the lenticular nucleus of the corpus striatum of the brain, the internal capsule remaining quite intact. In advanced cases the degeneration goes on to cavitation; but gross vascular disease and syphilitic changes are never found. There is no thrombosis of the vessels. Hepatic cirrhosis is an invariable concomitant, and of a severe grade; syphilis and alcohol can be excluded as its causes, and the cases occur in quite young people. The clinical symptoms of a fully-developed case are described as consisting of: bilateral involuntary movements, nearly always tremulous; weakness, spasticity, or hypertonicity, and eventually contracture of the skeletal musculature; dysarthria, or anarthria and dysphagia, and a degree of emotionalism, but without any sensory disturbances, without any true paralysis, and without any alteration in the cutaneous reflexes. If the abdominal reflexes are absent (apart from muscular rigidity) or the plantars are of extensor type, then the syndrome is no longer pure. It seems clear, says Dr. Wilson, that the disease is not due to a congenital or abiotrophic defect; the presumption is strong that it is acquired. There is evidence to show that the disease is toxic in origin, but none to suggest that the toxin is syphilitic. The nature of the toxin is speculative; the author thinks it is probably not microbic, and suggests that it may be elaborated in the liver. It has, he supposes, a specific action on the lenticular nucleus.

